# Transactional associations of child irritability and anxiety with parent psychological control in Taiwanese school‐aged children

**DOI:** 10.1002/jcv2.12192

**Published:** 2023-08-11

**Authors:** Ka Shu Lee, Eli R. Lebowitz, Wendy K. Silverman, Wan‐Ling Tseng

**Affiliations:** ^1^ Department of Experimental Psychology University of Oxford Oxford UK; ^2^ Yale Child Study Center Yale School of Medicine New Haven Connecticut USA

**Keywords:** anxiety, bidirectional influences, cross‐lagged panel model, irritability, parenting, psychological control

## Abstract

**Background:**

Child irritability and anxiety are associated with parent psychological control; yet their transactional relations over time are not well‐characterized at the within‐person level. Research addressing generalizability of past Western‐based literature in non‐Western, collectivist community samples is lacking.

**Methods:**

Sample comprised 285 children aged 8.8–11.4 years (145 girls; *M*age = 9.9 years, *SD* = 0.6) in Northern Taiwan. Participants were assessed at baseline (T1), 6‐month (T2), and 12‐month (T3) follow‐ups. Child irritability and anxiety symptoms were assessed using parent‐rated Child Behavior Checklist. Parent psychological control was assessed using the parent‐ and child‐rated Psychological Control Scale. Within‐person processes were specified using the random‐intercept cross‐lagged panel models.

**Results:**

Models showed that psychological control predicted increased child irritability when analyzing parenting data from parents and children. However, the lagged effect from psychological control to child anxiety was only seen in parent‐rated parenting data. We found limited evidence for a back‐and‐forth transactional pathway among constructs. Child irritability predicted increased child anxiety in all models.

**Conclusions:**

Directional effects from psychological control to child irritability and anxiety support parent‐involved interventions that prioritize collaborative parenting and positive reinforcement techniques. Future validations in combined clinical and typically developing samples and direct cross‐cultural comparisons are warranted.

**Key points**

- Child irritability and anxiety are associated with parent psychological control, but previous findings lacked specificity as to their within‐person relations over time and generalizability in non‐Western contexts.
- Psychological control provokes child irritability and anxiety over time.
- Child irritability also predicts anxiety.
- Negative outcomes of psychological control echo those found in Western samples.
- Directional effects support parent management training on collaborative parenting.

## INTRODUCTION

Early socioemotional symptoms and parenting jointly shape children's distress regulation (Kiff et al., 2011). This emphasis on the reciprocity between the parent and child has been applied to understanding the development of child irritability – a proneness to anger that may reach a clinical level (Stringaris et al., 2018; Vidal‐Ribas et al., 2016) – and anxiety, as their conceptual models have pointed to the role of parent‐child interactions and parenting in scaffolding emotion regulation beyond neurobiology and genetics (Strawn et al., 2021; Stringaris et al., 2018). Studies have identified various negative parenting behaviors associated with child irritability and anxiety, notably harsh and inconsistent discipline (Kircanski et al., 2019; Ravi et al., 2022; Lengua & Kovacs, 2005; Lengua, 2006; also see a meta‐analysis by McLeod et al., 2007 for anxiety). While these findings have inspired interventions focusing on improving parenting skills and parent‐child interactions, there is a call for more research on the mutual influences between child symptoms and parenting as they add specificity to treatment strategies (parent to child effects vs. child to parent effects) for meaningful and long‐term reduction in child symptoms (Pérez‐Edgar et al., 2021; also see an editorial on the lack of psychosocial interventions for irritability by Brotman & Kircanski, 2023).

### The role of parent psychological control in child irritability and anxiety

The current study focuses on parent psychological control— parent intrusions that involve shaming and constant invalidation of child behavior (e.g., irritable and anxious behaviors in this case), and withdrawal of parental love with little acknowledgment of child emotional distress (Barber, 1996). Psychological control is conceptually different from inconsistent discipline in that these tactics are consistent (yet maladaptive) responses with the aim to constrain the child's emotional experience and expression. Psychological control is *covert* and involves manipulation of the child's internal emotional and psychological experiences (e.g., to stop the child from throwing a tantrum by making them feel ashamed of being angry or irritable) (Choe et al., 2023), which is different from the disproportionate use of behavioral punishment seen in harsh parenting.

Studies have linked psychological control to child anxiety symptoms as being rejected and shamed by the parent are, in essence, potential threats to the child (Kiff et al., 2011). These tactics, in return, exacerbate child fearfulness and reinforce their tendency to be in a reactive state when responding to future anxiety‐inducing events (Bose et al., 2019; McLeod et al., 2007; van der Bruggen et al., 2008). Theoretically, it is suggested that psychological control prevents children from developing positive beliefs about their capabilities to cope with anxiety on their own (Barber, 1996). Clinically, psychological control has been demonstrated as a potential mediator between treatment and reduction in child anxiety in two randomized controlled trials where reduction in child anxiety following parent‐involved cognitive‐behavioral treatment was associated with lower psychological control at post‐treatment and 12 months later (Silverman et al., 2019, 2021).

For irritability, parent management training (PMT) has demonstrated some success in reducing child disruptive and irritable behaviors by teaching parents collaborative parenting (less parental control) and positive reinforcement techniques (Hawks et al., 2020; Linke et al., 2020). However, the association between psychological control and child irritability has not been empirically evaluated as they are often treated as secondary outcomes. From a threat processing perspective, shaming and the deprivation of parental warmth may signal the lack of safety and exacerbate the child's reactive responses to a distressing and often inescapable situation. On the other hand, psychological control can also be conceptualized in relation to frustrative nonreward—the angry‐frustrative state following the absence of expected reward that characterizes irritability (Kircanski et al., 2019; Vidal‐Ribas et al., 2016). Children tend to seek parental warmth and support when distressed. If their distress is instead responded with the parent's constant invalidation of the negative emotional experience and the potential threat of being rejected and shamed by the parent, the child's goal of seeking parenting support to help regulate the distress is blocked, which may drive further frustration (Lengua, 2006; Nelson & Crick, 2004). Relatedly, past studies on reactive aggression, a maladaptive response to threats commonly observed in irritable children (Kircanski et al., 2019), suggested its link with parent psychological control (Nelson & Crick, 2004; Nelson et al., 2006).

### Parent psychological control in non‐western contexts

At the contextual level, prior research has found generalizable evidence for the negative developmental outcomes of psychological control in non‐Western contexts (e.g., child externalizing and internalizing symptoms, including anxiety in Olsen et al., 2002 conducted in Voronezh, Russia and Beijing; child aggressive behaviors in Nelson et al., 2006 conducted in Beijing; and in Chinese American families in Yu et al., 2019) (also see a meta‐analysis by Chyung et al., 2022: *r* = 0.29 in individualist cultures vs. *r* = 0.32 in collectivist cultures for child depression, and *r* = 0.20 in individualist cultures vs. *r* = 0.29 in collectivist cultures for child anxiety). However, studies on the reciprocal relations between our constructs of interest are scarce (Settipani et al., 2013; Silverman et al., 2019, Silverman et al., 2021 – all of which focused on child anxiety in Western samples), and that extrapolating from what has been known in Western samples is untenable due to potential cultural differences (Malti & Cheah., 2021). Here, we address this issue by sampling school children and their parents in Taiwan, a collectivist culture where interdependence is valued and the child expression of fear and anger is considered socially‐inappropriate and detrimental to interpersonal relationships (Cho et al., 2021; Nelson et al., 2006). These socialization goals contrast with those in most Western and individualist cultures where there is a greater tolerance toward the child expression of negative emotions, whereas Taiwanese parents may tend to engage in parental control behaviors such as psychological control to manage the child's overt expression of socially‐disengaging emotions (Jose et al., 2000; Louie et al., 2013). An investigation of these intertwined processes in Taiwan would serve as a test of generalizability for what has been found in Western samples so far.

### Testing within‐person transactional pathways

The cross‐lagged panel model (CLPM) provides a theory‐driven framework for testing these reciprocal, intertwined relations between child irritability, anxiety, and psychological control as it simultaneously accounts for the contemporaneous, autoregressive, and cross‐lagged paths among constructs (Curran & Bauer, 2011). Prior to its wide application in the field, only few past studies have demonstrated the prospective, bidirectional relations between these constructs yet in separate regression models. Two community studies (ages 8–12) that sampled annually over two (Lengua & Kovacs, 2005: *N* = 92) and 3 years (Lengua, 2006: *N* = 190) have found that, using composite mother‐ and child‐rated measures, child irritability predicted *increases* in inconsistent discipline, whereas child fearfulness predicted *decreases* in inconsistent discipline and rejection. Conversely, inconsistent discipline and rejection also predicted *increases* in child irritability and fearfulness. These findings, however, do not necessarily test a transactional pathway that negative parenting behaviors (psychological control in our case) may elicit more child irritability and anxiety, which in turn may evoke *even more* negative parenting behaviors over time (or in reverse, child‐to‐parent then back to child).

Crucially, past CLPM research has only considered the relations between child anxiety and psychological control at the between‐person level (Settipani et al., 2013; Silverman et al., 2019, 2021). Parallel work on child irritability is not available. This conceptualization implies that these constructs are time‐invariant and trait‐like, which may not be the case for most developmental changes (Hamaker et al., 2015). Although informative regarding the overall average development across individuals, the traditional CLPM overlooks parent‐child processes in terms of their deviations from the dyad's own expected level(s) of parenting behavior/child symptom at the within‐person level (Curran & Bauer, 2011; Hamaker et al., 2015). Interpreting between‐person processes alone can be problematic as the correlation between developmental constructs may vary in strength and directionality for within‐person changes (Hamaker et al., 2015; Mulder & Hamaker, 2022).

### Current study

**FIGURE 1:**
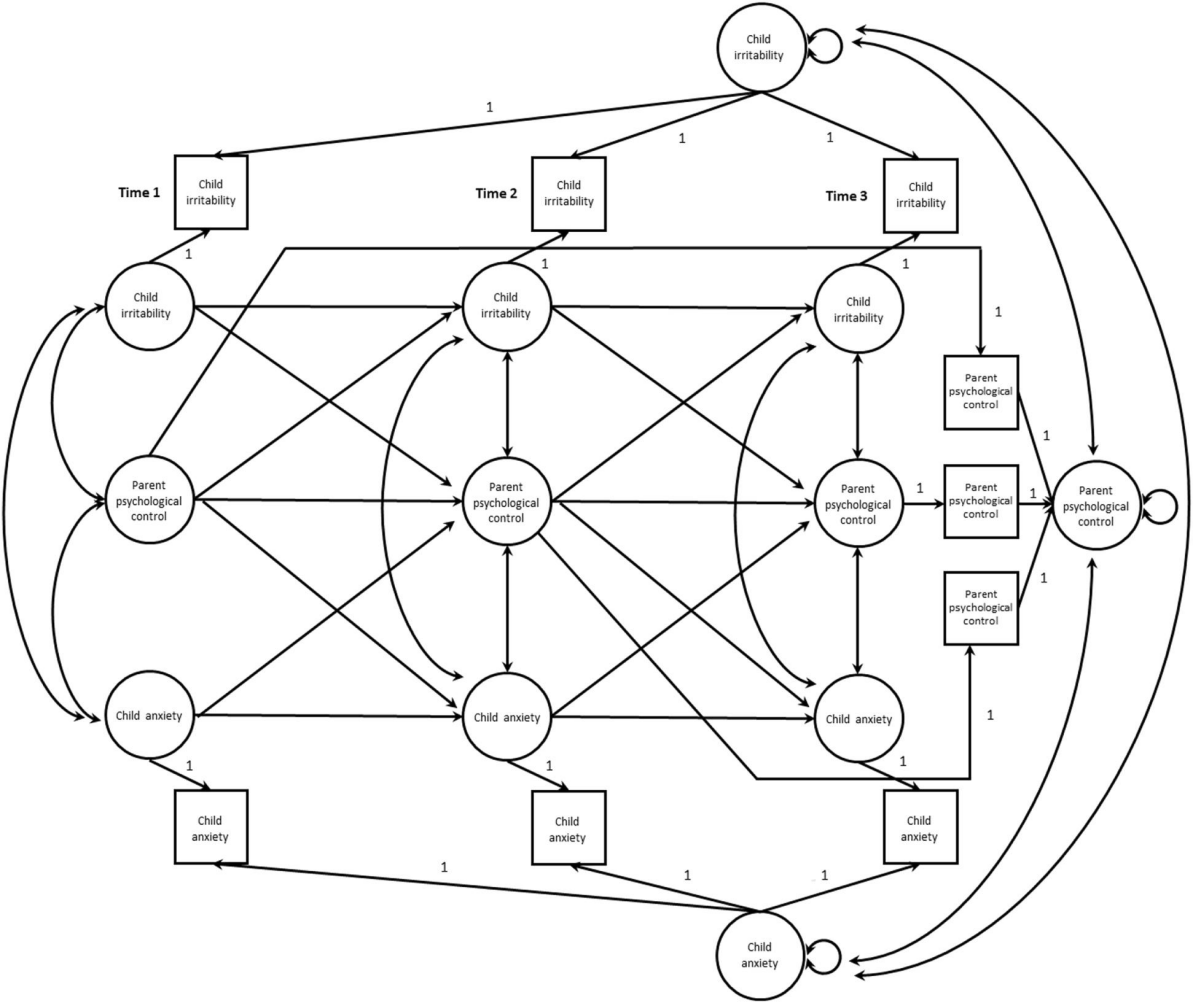
Hypothesized transactional model of child irritability, child anxiety, and parent psychological control. The random‐intercept cross‐lagged panel model (RI‐CLPM) disentangles between‐person level changes (from the grand mean) from within‐person level changes (from one's expected scores). As an example, the cross‐lagged paths between parent psychological control and child irritability represent how a parent's increased tendency to practice psychological control relative to their own expected tendency, is related to a child's elevated level of irritability relative to their own expected level of irritability at the next timepoint. The autoregressive paths represent the carry‐over effects of a construct over time. The stable, trait‐like variances of these constructs are captured by the latent between‐person random intercepts, denoted by the paths constrained to 1. See Mulder and Hamaker (2021) for setting up the RI‐CLPM.

Taken together, using three waves of data collected over 12 months, we examined the transactional relations between child irritability, anxiety, and parent psychological control via the random‐intercept CLPM (RI‐CLPM; Figure 1) (Hamaker et al., 2015). The paths that we tested were child irritability and anxiety at Time 1 (T1) → psychological control at Time 2 (T2) → child irritability and anxiety at Time 3 (T3); as well as psychological control at T1 → child irritability and anxiety at T2 → psychological control at T3. This new variant of the CLPM captures stable, trait‐like between‐person changes in these constructs as random intercepts while modeling the within‐person processes in a transactional manner. The three assessments were 6 months apart to test if these within‐person relations emerge in a shorter timeframe than those in previous studies (Lengua, 2006; Lengua & Kovacs, 2005; also see a paper on sampling interval by Dormann & Griffin, 2015). We collected parent‐rated child irritability and anxiety symptoms. Since parenting is a dyadic process, we collected parent‐ and child‐rated psychological control, which offer a cross‐informant perspective when assessing negative parenting qualities that are often under‐rated by parents (Nielson et al., 2006; Yu et al., 2015).

Based on non‐Western findings (Nelson et al., 2006; Olsen et al., 2002) and the recent trials on child anxiety (Silverman et al., 2019, 2021), we hypothesized that there would be positive transactional associations between child irritability and anxiety and parent psychological control in this Taiwanese school‐aged sample.

## METHOD

### Participants

This study is part of a 12‐month prospective school‐based study in Northern Taiwan. Assessment took place every 6 months at three timepoints (T1, T2, T3). Briefly, the original study included 739 children (239 fourth graders and 500 fifth graders; 353 girls). Parents and children provided written informed consent to participate. Full recruitment details and overall sample characteristics of the original study are described elsewhere (Tseng et al., 2013, 2014). For the purpose of this study, 285 children (141 fourth graders and 144 fifth graders; 145 girls) were selected on the basis that they had complete information on T1 irritability and T1 anxiety, and T2 or T3 parent‐ and child‐rated psychological control. This allows for testing the cross‐lagged paths of interest, with <40% missing data across all study variables at each timepoint (range = 0%–38.5%) for subsequent missing data imputation (Jakobsen et al., 2017; van Ginkel et al., 2020). No significant differences in sample characteristics and study variables between the analytic and excluded samples were found, except for children's age (Table S1).

The mean age of the analytic sample was 9.9 years at baseline (*SD* = 0.6; range = 8.8–11.4 years). Most children lived with both parents (88.4%), were from middle‐income families (1524 USD to 3049 USD per month; 46.7%), and had mothers who attained college or beyond (46.3%) (Table 1). This study was approved by the Institutional Review Board at the last author's university at the time of the study. Measures were translated into traditional Chinese and back translated into English by a native speaker of both languages. All children received a stationery set for participating in the study.

**TABLE 1:** Sample characteristics (*N* = 285).

	*n/*Mean	%/*SD*
Child's age (years; continuous)	9.9	0.6
Child's age (grade level), *n* (%)		
4^th^ grade	141	49.5
5^th^ grade	144	50.5
Mother's age (years; continuous)	40.1	4.7
Child's sex (female), *n* (%)	145	50.9
Living arrangement, *n* (%)		
Living with both parents	252	88.4
Single‐parent family and other	33	11.6
Mother's education level, *n* (%)		
Junior high or below	41	14.6
High school	110	39.1
College or above	130	46.3
Monthly family income, *n* (%)		
Low income (below 1524 USD)	98	35.8
Mid income (1524 to 3049 USD)	128	46.7
High income (above 3049 USD)	48	17.5
Informant, *n* (%)		
Mothers	201	70.5
Fathers	66	23.2
Other primary caretakers	18	6.3

*Note*: Other primary caretakers are relatives of the child.

### Missing data

A missing data analysis was performed in the original sample, and it was found that the missingness of our key study variables (irritability, anxiety, and parent psychological control) was associated with children who were fifth graders, from low‐income families, with mothers who attained high school education only, and lived with both parents. These variables were hence included as covariates in the modeling procedures.

To increase power for our analyses, item‐level imputation was done to fill in missing data in the analytic sample (*N* = 285) using missForest in R, a random‐forest based iterated imputation approach (Stekhoven, 2013) (see Appendix S1). No significant differences in distributions and statistical properties were found for all study variables before and after imputation (*t*s = −1.32–1.07, *p*s = 0.19–1) (Figure S1), suggesting robust learning of seed data (normalized root mean squared error = 0.00002 and proportion of falsely classified = 0.09) and successful imputation of missing data in the absence of bias.

### Measures

#### Child symptom measures completed by parents

*Child irritability.* Parents rated their children's irritability symptoms using three items from the *Child Behavior Checklist* (CBCL; Achenbach, 1991), namely “*86. stubborn, sullen or irritable*,” “*87. sudden changes in mood or feelings,*”, and “*95. temper tantrums or hot temper*” (0 = not true; 1 = somewhat true; 2 = very true or often true). The mean scores of the three items were analyzed (range = 0–2). The CBCL irritability items show good internal consistency (Tseng et al., 2017; Wiggins et al., 2014), a single factor structure (Wiggins et al., 2014), excellent test‐retest reliability, and are correlated with the Affective Reactivity Index (ARI; Stringaris et al., 2012) (*rs* = 0.26–0.64) (Tseng, 2020; Tseng et al., 2017), and capture an adequate to good amount of individual differences in the latent irritability trait (alpha ∼0.80) (Dougherty et al., 2021). In this sample, the internal consistency (Cronbach's alpha) at each timepoint was 0.80, 0.85, and 0.79.

*Child anxiety.* Parents rated their child's anxiety symptoms using the anxiety items on the CBCL Anxious/Depressed subscale (Achenbach, 1991; Kendall et al., 2007). The eight items were rated on a three‐point Likert scale (0 = not true; 1 = somewhat true; 2 = very true or often true). The mean scores of the anxiety items were analyzed (range = 0–2). The CBCL anxiety items show better discriminant validity and comparable construct validity relative to other parent‐rated child anxiety measures (Kendall et al., 2007) and is widely used in child psychiatric research in Taiwan (e.g., Chen et al., 2017). Internal consistency in the current sample at each timepoint was 0.77, 0.81, and 0.82.

#### Parenting measure completed by parents and children

*Parent psychological control.* Parents rated their self‐perceived level and frequency of psychological control (0 = not at all; 5 = always) using the validated Chinese version of the Psychological Control Scale (Nelson et al., 2006; Yu et al., 2015). The scale comprises 10 items assessing two key aspects of psychological control, namely love withdrawal (five items, e.g., “*Avoid looking at my child when my child has disappointed me*”) and guilt induction (five items, e.g., “*Making my child aware of how much I sacrifice or do for him/her*”). Children also rated on a child version of the measure, with identical items reworded as “… *my parents*…” to assess children's perception of their parents' psychological control parenting. The within‐time correlations between parent‐ and child‐rated scores were small to moderate (*r*s = 0.27, 0.26, and 0.24 at T1–T3), and parents on average rated lower psychological control scores than children at each timepoint (*t*s = −18.23, −14.46, and −13.25, *p*s < 0.001). We therefore analyzed the mean parent ratings and mean child ratings (range = 1–5) in separate models. Internal consistency in the current sample at each timepoint was 0.80, 0.88, and 0.91 for parent ratings, and 0.79, 0.84, and 0.85 for child ratings.

### Statistical analyses

We conducted random‐intercept cross‐lagged panel model (RI‐CLPM) using Mplus version 8 with maximum likelihood parameter estimates that are robust to non‐normality (Muthén & Muthén, 1998‐2017). All other statistical analyses were performed in R (R Core Team, 2020). A series of bivariate correlation analyses was first conducted to describe the associations of all study variables across three timepoints. Next, we used the RI‐CLPM to estimate the cross‐lagged paths, autoregressive and contemporaneous relations between the study variables (Hamaker et al., 2015). We conducted two separate models using parenting data from parents and children. Random‐intercept cross‐lagged panel model uniquely disentangles stable, trait‐like between‐person differences in the developmental constructs from within‐person variances of the same constructs at each timepoint (Curran & Bauer, 2011; Hamaker et al., 2015; see Appendix S2). Informed by the missing data analysis, children's grade level, maternal education, living arrangement, and family income were regressed on the random intercepts of the study variables (Mulder & Hamaker, 2021).

Model fit was indexed by Comparative Fit Index (CFI) (≥0.95), Root Mean Square Error of Approximation (RMSEA) (≤0.05), and Standardized Root Mean Square Residual (SRMR) (≤0.08) (Hu & Bentler, 1999; McDonald & Ho, 2002). Alternatively, CFI ≥0.90, and RMSEA ≤0.10 also indicate adequate fit (McDonald & Ho, 2002). Model Chi‐square (χ^2^) was considered a secondary fit index as sample sizes may bias estimates. Standardized estimates were reported. Modification indices were used to improve the fit of the hypothesized model, and it was suggested that the cross‐lagged paths between irritability and anxiety (and vice versa) should also be estimated and were hence added to the final models. All statistical tests were significant at alpha = 0.05, two‐tailed.

## RESULTS

### Descriptive statistics

Table 2 presents the means, standard deviations (*SD*s), and bivariate correlations of the study variables across all timepoints. Numerous positive contemporaneous and prospective associations were observed among variables, providing initial support for testing the transactional relations.

**TABLE 2:** Descriptive statistics and correlations between study variables.

		1	2	3	4	5	6	7	8	9	10	11	12	13	Mean	*SD*	Min	Max
1	Age	‐‐													9.9	0.6	8.8	11.4
2	Sex	−0.02	‐‐												‐‐	‐‐	‐‐	‐‐
3	IRR1	−0.03	−0.1	‐‐											0.27	0.40	0	2
4	IRR2	−0.07	−0.02	0.48***	‐‐										0.23	0.39	0	1.7
5	IRR3	−0.12*	−0.02	0.45***	0.45***	‐‐									0.23	0.39	0	2
6	ANX1	−0.06	0.02	0.47***	0.37***	0.31**	‐‐								0.24	0.29	0	1.4
7	ANX2	0	0.02	0.35***	0.56***	0.30***	0.47***	‐‐							0.19	0.26	0	1
8	ANX3	−0.09	0.05	0.38***	0.36***	0.57***	0.55***	0.44***	‐‐						0.19	0.28	0	1.8
9	PPC1	−0.02	−0.05	0.33***	0.31***	0.37***	0.32***	0.27***	0.29***	‐‐					1.89	0.50	1	3.7
10	PPC2	−0.01	−0.06	0.27***	0.33***	0.32***	0.29***	0.25***	0.28***	0.55***	‐‐				1.90	0.58	1	5
11	PPC3	−0.03	−0.1	0.29***	0.33***	0.3***	0.34***	0.27***	0.28***	0.55***	0.75***	‐‐			1.89	0.72	1	4.8
12	CPC1	0.05	0.03	0.16**	0.09	0.19**	0.03	0.04	0.01	0.27***	0.17**	0.21***	‐‐		2.43	0.78	1	5
13	CPC2	0	0.01	0.18**	0.09	0.26***	0.08	0.03	0.11	0.28***	0.26***	0.22***	0.53***	‐‐	2.40	0.83	1	5
14	CPC3	0.08	0.01	0.12*	0.03	0.14*	0.06	0	0.08	0.26***	0.18**	0.24***	0.49***	0.58***	2.45	0.87	1	5

Abbreviations: ANX, child anxiety; CPC, child‐rated parent psychological control; IRR, child irritability; PPC, parent‐rated parent psychological control.

*p ≤ 0.05, **p ≤ 0.01, ***p ≤ 0.001.

### Transactional models of irritability and anxiety over time

We first present models using parenting data from parents, and then report if results converge or differ when using parenting data from children.

#### Parenting data from parents

**FIGURE 2:**
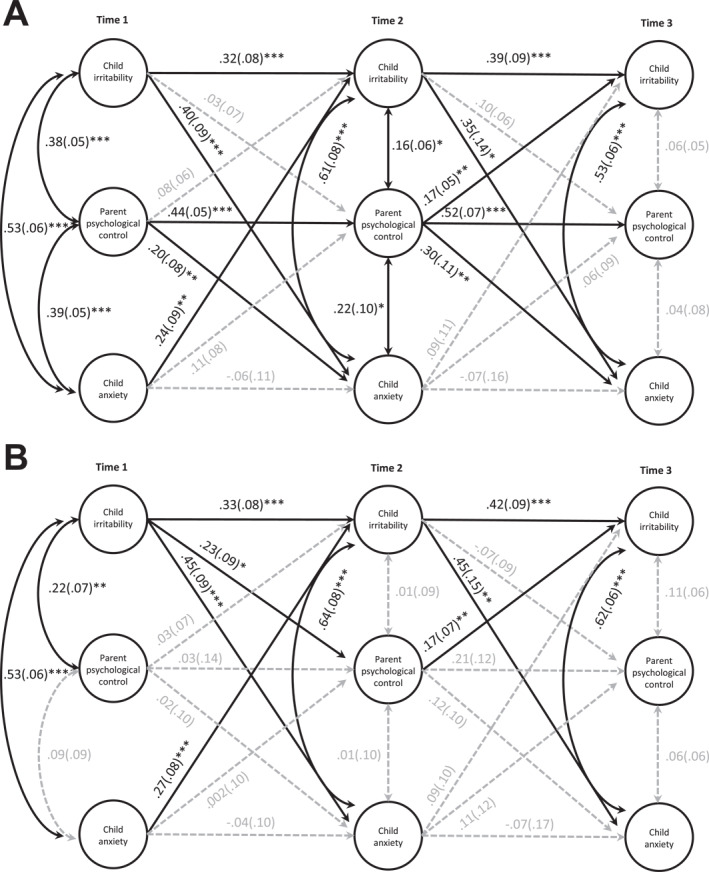
Transactional associations between child irritability, child anxiety, and parent psychological control. (A) Parenting data from parents. The variances of the random intercepts for child irritability and parent‐rated psychological control were constrained to zero. (B) Parenting data from children. Only the variances of the random intercept for child irritability were constrained to zero. Significant paths are denoted by solid black lines; non‐significant paths are denoted by dashed gray lines. Standardized estimates (standard errors) are shown. To avoid clutter, the paths estimating the between‐person random intercepts were included in the model setup but are not visualized here. The cross‐lagged paths between child irritability and anxiety were included based on modification indices. *p ≤ 0.05, **p ≤ 0.01, ***p ≤ 0.001.

In the attempt to extract between‐person variances for all study variables, the initial model indicated that these latent variables for irritability and parent psychological control were not significant, meaning that there was a lack of variances accounting for the stable, trait‐like characteristics of the two constructs (Mulder & Hamaker, 2021). By constraining their variances (and covariances) to zero, the resulting model showed good fit to the model, χ^2^(32) = 62.54, *p* = 0.001, CFI = 0.96, RMSEA = 0.06 [0.04, 0.08], and SRMR = 0.05 (Figure 2A). Specifically, T1 psychological control predicted increased T2 anxiety (Beta = 0.20, SE = 0.08, *p* = 0.01); T2 psychological control also predicted increased T3 irritability (Beta = 0.17, SE = 0.05, *p* = 0.002) and T3 anxiety (Beta = 0.30, SE = 0.11, *p* = 0.004). A transactional path was found between anxiety and irritability where T1 anxiety predicted increased T2 irritability (Beta = 0.24, SE = 0.09, *p* = 0.01), which then predicted increased T3 anxiety (Beta = 0.35, SE = 0.14, *p* = 0.01). Further, T1 irritability predicted increased T2 anxiety (Beta = 0.40, SE = 0.09, *p* < 0.001). The autoregressive paths for irritability (Betas = 0.32 and 0.39) and psychological control (Betas = 0.44 and 0.52) suggest that there were significant carry‐over effects (i.e., stability) within these constructs over time (*p*s < 0.001).

#### Parenting data from children

While the variances of the random intercept for irritability remained constrained to zero, the latent variable accounting for stable, trait‐like features in child‐rated parent psychological control was significant. This suggests that such between‐person variances were more prevalent in children's perception of parenting than their caregivers' perception as analyzed above. Models analyzing *child‐rated* psychological control revealed largely consistent results. Specifically, the model demonstrated good fit, χ^2^(31) = 49.13, *p* = 0.02, CFI = 0.97, RMSEA = 0.05 [0.02, 0.07], SRMR = 0.04 (Figure 2B). A transactional path was found between irritability and psychological control where T1 irritability predicted increased T2 psychological control (Beta = 0.23, SE = 0.09, *p* = 0.02), which then predicted increased T3 irritability (Beta = 0.17, SE = 0.07, *p* = 0.01), the latter of which was consistent with the parent‐rated data above. The same cross‐lagged path between T1 irritability and T2 anxiety (Beta = 0.45, SE = 0.09, *p* < 0.001) and the transactional path between T1 anxiety and T2 irritability (Beta = 0.27, SE = 0.08, *p* = 0.001) and back to T3 anxiety (Beta = 0.45, SE = 0.15, *p* = 0.003) were found in the child‐rated data. Only the autoregressive paths for irritability were significant over time (Betas = 0.33 and 0.42, *p*s < 0.001).

### Supplementary analyses

To examine the specificity of our main findings, the models were re‐run with further adjustments for parent sad mood and child depressive and attention deficit hyperactivity disorder (ADHD) symptoms at baseline, as these co‐occurring emotional and behavioral problems may explain some of the variances between child irritability and anxiety and psychological control (see Appendix S3). When analyzing parenting data from parents, results were largely consistent, except that the transactional path along T1 anxiety → T2 irritability → T3 anxiety was non‐significant (Figure S2A). For child‐rated data, the transactional path along T1 irritability → T2 psychological control → T3 irritability became marginally significant (Figure S2B). The cross‐lagged paths from T1 irritability to T2 anxiety and from T2 irritability to T3 anxiety remained significant.

## DISCUSSION

This is the first study that simultaneously tests the within‐person lagged effects of child irritability and anxiety with parent psychological control in Taiwanese school‐aged children, who are exposed to a collectivist cultural context. Three novel findings are: (a) the transactional path where child irritability at T1 predicted increased psychological control at T2, which then provoked further increase in child irritability at T3 when analyzing child‐rated parenting data (although these relations became marginally significant when further controlling for parent and child characteristics); (b) the lagged paths where psychological control predicted increases in child irritability and anxiety; and (c) the lagged paths where child irritability predicted increases in child anxiety.

Despite the intention to discipline children to behave in socially‐appropriate ways, the lagged paths from psychological control to child irritability suggest that shaming and love withdrawal tactics yet result in higher levels of irritability in the future (Kiff et al., 2011). This highlights the possibility that, besides the common focus on threat perception, psychological control can also be viewed as a form of frustrative nonreward where a child's expectation for parental support at times of distress is not fulfilled (such as being responded with the parent's expression of shame and disappointment instead), which exacerbates child frustration (Kircanski et al., 2019; Vidal‐Ribas et al., 2016). This latter focus on the child's need and tendency to seek parental support to help regulate distress is evident in PMT that promotes parental warmth and collaborative parenting to reduce irritability (e.g., Hawks et al., 2020). However, we found no robust evidence for the presence of transactional paths between psychological control and child irritability, meaning that increased psychological control or elevated child irritability alone does not necessarily lead to a back‐and‐forth maintaining mechanism over time. Some potential confounds underlying their reciprocity may be parent sad mood and child depressive and ADHD symptoms as informed by our supplementary models. In particular, maternal depression was found to be transactionally associated with child irritability over 9 years (Wiggins et al., 2014, *N* = 4898), which may make these mothers more prone to intrusive parenting behaviors and fewer expressions of parental love (Goodman et al., 2011; Lovejoy et al., 2000). Clinically, this highlights that reducing parental control may be one intervention target to be considered alongside other developmental factors of persistent irritability (Kircanski et al., 2019; Vidal‐Ribas et al., 2016). Alternatively, a longer study period or the collection of time‐series data (which intensively captures moment‐to‐moment parent‐child interactions) might allow these within‐person transactional paths to emerge.

The commonly‐reported linkage between psychological control and anxiety was only present in our parent‐rated data. Some possible explanations may be that the Taiwanese parents might have under‐reported their psychological control tendency due to a high awareness of social desirability in the collectivist culture (Chen et al., 2017), or that these children in fact have a different understanding of psychological control from their parents (Choe et al., 2023, also see a discussion on multi‐informant assessments by De Los Reyes et al., 2015). When comparing to past between‐person findings leveraged from parent‐rated parenting data, our lagged paths highlight a parent to child effect from parent psychological control to child anxiety (Settipani et al., 2013) but not a child to parent effect from child anxiety to parent psychological control (Silverman et al., 2019, 2021). Besides these past studies' different focus on between‐person effects and clinical samples (which may hence speak to a greater child impact on their parents), the inconsistency may be due to confounding variances from a potential effect between psychological control and irritability, which was not measured in the studies.

Studies on both clinical and healthy samples have found that co‐occurring irritability is associated with more severe anxiety symptoms and related impairments (e.g., Shimshoni et al., 2020; Stoddard et al., 2014). Our findings highlight a consistent lagged effect from irritability to increased anxiety across our main and supplementary models; a reverse anxiety to irritability effect or a back‐and‐forth transactional pathway, however, is non‐significant in supplementary models further controlling for parent and child emotional and behavioral problems. This irritability to anxiety effect echoes a number of large longitudinal studies that found irritability to be predictive of future anxiety symptoms and disorders (e.g., Copeland et al., 2014; Stringaris et al., 2009). For instance, a community study of 631 adolescents (mean age = 13.8 years) found that irritability was predictive of general anxiety disorder 20 years later even after controlling for other socioemotional symptoms (Stringaris et al., 2009). Here, we demonstrate that this directional effect can be observed at a much shorter timescale (6 months), suggesting that irritability may be a precursor to the development of anxiety. The symptomatology that underlies this linkage requires further research, but insights from a smartphone experience sampling study (*N* = 152, including 34 with disruptive mood dysregulation disorder and 29 with anxiety disorder; mean age = 12.3 years) found that frustration may play a central role as it predicted more between‐ and within‐person mood changes and worry between prompts (Tseng et al., 2023).

Not least of all, sampling Taiwanese children and parents gives us the unique opportunity to address the generalizability of the finding that psychological control is associated with negative developmental outcomes in both Western (e.g., Settipani et al., 2013; Silverman et al., 2019; Silverman et al., 2021) and non‐Western contexts. Parents tend to engage in parenting behaviors that conform to their cultural values. Taiwanese parents may value interdependence more, and thus engage in parenting behaviors that constrain the expression of socially‐disengaging emotions such as anger and anxiety to foster children's interpersonal relationships. Western parents, on the other hand, may value independence more and encourage children's emotional expressions as an expression of individuality (Cho et al., 2021; Yu et al., 2019). A critical next step is to conduct direct cross‐cultural comparisons (e.g., Nelson et al., 2006; Olsen et al., 2002), testing if the expression of anger and frustration is highly discouraged in collectivist cultures such as in Taiwan, thereby potentially contributing to a stronger lagged effect between child irritability and parent psychological control than that in Western samples (Chyung et al., 2022).

### Limitations

Several limitations are noted. Although construct and convergent validity of the CBCL irritability items and other measures of irritability have been well‐documented (such as the ARI, see Methods), the CBCL irritability phenotype corresponds to the temperamental model of irritability, which views mood lability (low awareness of abrupt and drastic mood changes) as an irritability symptom (Stringaris & Goodman, 2009; Vogel et al., 2022). Note that the ARI was not available at the time of data collection, whereas the CBCL items were considered the best available measure of irritability at the time. Future validations using dedicated clinical measures of irritability are warranted. Moreover, our findings are drawn from a non‐clinical sample with parents who generally rated low levels of child irritability and anxiety symptoms. Future studies capturing the full range of symptom severity, such as through a mixed sample comprising clinical, at‐risk, and typically‐developing participants, are necessary to clarify these intertwined relations. A more socioeconomically diverse sample may also inform the generalizability of our findings to less‐resourced families. Finally, it is possible that psychological control is predisposed to the shared genetic link between parent and child irritability and anxiety. Irritable and/or anxious parents may be more likely to exert control over their children's behavior as a strategy to manage their own irritability and anxiety in parenting situations, which are then modeled by their children. Twin studies and longitudinal designs adjusting for the polygenic scores of irritability and anxiety symptoms may be useful (Shewark et al., 2021).

### Clinical implications and conclusions

Limitations aside, our findings support psychological control as a promising target for reducing child anxiety as demonstrated in previous randomized controlled trials (Silverman et al., 2019, 2021) and disruptive and irritable behaviors as demonstrated in PMT‐inspired interventions on collaborative parenting (less parental control and more positive reinforcements) (Hawks et al., 2020; Linke et al., 2020). Better understanding of the directionality between parent and child behaviors at the within‐ and between‐person levels may guide more cost‐effective and tailored intervention strategies (Hamaker et al., 2015). When working with individual parent‐child dyads (within‐person level), clinicians may focus on addressing specific parenting concerns and replacing intrusive tactics (shaming and love withdrawal) with positive parenting techniques for managing child irritable and/or anxious behaviors. Between‐person effects may point to the potential of group‐ and school‐based parent‐involved interventions, such as psychoeducation on the drawbacks of psychological control. Although our lagged effects echo those reported in Western samples, the underlying motivation to practice parental control may be different among Taiwanese parents (Malti & Cheah, 2021). This may again stem from the collectivist emphasis on interpersonal relationships and emotional restraint as noted above. Therefore, clinicians are encouraged to attend to the sociocultural meaning of childrearing practices and consider parenting skills that appreciate these culturally‐valued qualities. Rapid emergence of these lagged influences underscores the importance of timely assessment and intervention.

## AUTHOR CONTRIBUTIONS

K.S.L. planned and conducted the analyses, interpreted the data, drafted and revised important intellectual content of the manuscript. E.R.L. and W.K.S. interpreted the data, revised important intellectual content of the manuscript, and provided supervision. W.‐L.T. conceptualized and designed the study, acquired the data, interpreted the data, revised important intellectual content of the manuscript, and provided primary supervision. All authors read and approved the version to be published, and agreed to be accountable for all aspects of the work in ensuring that questions related to the accuracy or integrity of any part of the work are appropriately investigated and resolved.

## CONFLICT OF INTEREST STATEMENT

The authors have declared that they have no competing or potential conflicts of interest.

## ETHICAL CONSIDERATIONS

This study was approved by the Institutional Review Board (#0802S26261) at University of Minnesota.

## Supporting information

Supplementary Material

## Data Availability

The data that support the findings of this study are available from the corresponding author upon reasonable request. Supplementary materials are available via Open Science Framework: DOI 10.17605/OSF.IO/VM52Z.
