# Supplementary material for: Transactional associations of child irritability and anxiety with parent psychological control in Taiwanese school‐aged children
Source: JCPP Adv. 2023 Aug 11;3(4):e12192. doi: 10.1002/jcv2.12192 (PMC10694542; doi:10.1002/jcv2.12192)
Supplement: Supplementary file 1 — Supplementary Material [file JCV2-3-e12192-s001.docx]

**Supporting Information**

Transactional associations of child irritability and anxiety with

parent psychological control in Taiwanese school-aged children

**Appendix S1. Study Sample and Missing Data Imputation**

The current sample (*N*=285) was selected from the original sample (*N*=739) based on those with complete information for parent-reported child irritability and anxiety at T1, and parent- and child-reported parent psychological control at follow-up (T2 or T3). The percentages of missing data of the current sample across timepoints were 0-34% at T1, 20-39% at T2, and 14-30% at T3. The higher proportion of missing data at T2 and T3 was largely due to one of the participating schools dropping out at T2 (69.55% of the original sample remained at T3; see Tseng et al., 2014), and from the psychological control measures, which were relatively lengthy as compared to other assessments and placed at the end of the survey. Nonetheless, statistical comparisons between the analytic and excluded samples suggested no significant differences in their sample characteristics and study variables across timepoints, except for a small difference in children’s age (Table S1).

To increase the number of observations and statistical power, we performed imputation using the missForest package in R (Stekhoven, 2013). Relative to other imputation methods, the learning algorithm of missForest is a random-forest based imputation approach that makes no assumption of normality/linearity/interaction among data, and has been shown to be particularly suitable for imputing longitudinal data sets with both categorical and continuous variables with desirable estimation results (Enders, 2013; Shah et al., 2014; Hong & Lynn, 2020). Studies have demonstrated that missing data imputations are effective to increase sample size and power for the analysis, granted that the proportions of missing data are not too large (i.e., > 40%; Jakobsen et al., 2017; van Ginkel et al., 2020). Random-forest based methods are also more robust to assumptions of missingness (random/non-random) than conventional regression-based imputations (Hong & Lynn, 2020; Waljee et al., 2013). Following these various guidelines would allow us to feed the algorithm on data that are readily available for unbiased sampling of seed data and iterated learning of statistical features *across* the entire landscape of dataset. We therefore decided to impute the missing data within the analytic sample (*N*=285, with complete information provided for our key variables of interest across timepoints as mentioned) in which the proportions of missing data among the study variables were within the recommended threshold of 40%, ensuring imputed data quality. Data imputation based on the full sample (*N*=739) with missing data above the 40% threshold would risk biasing the initial sampling of seed data, compromising not only the subsequent algorithmic learning of statistical characteristics in the current data, but also data analysis, and interpretability and reproducibility of those modeling results (Enders, 2013; van Ginkel et al., 2020; Jakobsen et al., 2017; Stekhoven, 2013). The current sample size was in line with recommendations for prospective developmental research and provides sufficiently large data seed for algorithmic sampling and iterated predictions (Enders, 2013). Statistical comparisons indicated no significant differences in means and SDs between all study variables before and after imputation (*t*s=-1.32–1.07, *p*s=.19–1). Density plots displaying the distributions of key variables of each dataset at each time point were indeed highly comparable (Figure S1), which further confirmed an improvement in data completeness after imputation.

**Appendix S2. Random-Intercept Cross-Lagged Panel Modeling (RI-CLPM)**

The standard cross-lagged panel modeling (CLPM) has been criticized for not adequately reflect the within-person transactional relationships over time, which is crucial to understanding the developmental process associated with child symptoms and parent behaviors within *individual* parent-child dyads (Curran & Bauer, 2011; Hamaker et al., 2015; Sameroff, 2000). Specifically, the RI-CLPM adopts a latent variable approach to individual mean centering and disentangle time-invariant, trait-like between-person variances in the study constructs across time points from within-person variances in the same constructs at each time point. This unique modeling approach hence produces stability and cross-lagged path estimates reflective of how a particular individual or parent-child dyad varies in the levels of the same and/or different constructs and predicts within-person changes of those constructs over time (Hamaker et al., 2015). At a between-person level, the correlations between the latent between-person constructs reflect if parent-child dyads that rank higher/lower on a construct as compared to others *on average* across time points also rank higher/lower overall on the other construct (Hamaker et al., 2015). In this study, these modeling constructs refer to child irritability, child anxiety, and parent psychological control. We presented in the main text modeling results from surveys completed by the parents (93.7%), as well as other primary caretakers who were relatives of the child, such as grandparents, aunts, and uncles (6.3%). Results remained unchanged by restricting to parent-completed surveys only.

**Appendix S3. Transactional Models with Further Adjustments**

The random-intercept models were re-run with further adjustments for parent sad mood and child depressive and attention deficit hyperactivity disorder (ADHD) symptoms at baseline. Parent sad mood was measured by the item “How often do you feel down (depressed) or feel like crying” (0=never; 3=always), which was designed based on items 1 and 10 on the Beck’s Depression Inventory (Beck et al., 1988). The raw scores were analyzed (mean=0.72, *SD*=0.52) For child depressive symptoms, the mean raw scores of the depression items on the CBCL Anxious/Depressed subscale were used (Achenbach, 1991) (mean=0.18, *SD*=0.27). For child ADHD symptoms, the mean raw scores of the parent-reported Swanson, Nolan, and Pelham, version IV scale (SNAP-IV) (Gau et al., 2008; Swanson, 1992) were used (mean=0.66, *SD*=0.42). These variables were treated as covariates and regressed on the random intercepts of the key study variables (i.e., child irritability, child anxiety, and parent psychological control) (Mulder & Hamaker, 2021).

Results suggested that the model revealed largely consistent results when analyzing parenting data from parents, χ^2^(50)=74.50, *p*=.02, CFI=.97, RMSEA=.04 [.02, .06], SRMR=.05, except that the transactional path from T1 anxiety 🡪 T2 irritability 🡪 T3 anxiety was non-significant (Figure S2A). When analyzing parenting data from children, χ^2^(49)=62.88, *p*=.09, CFI=.98, RMSEA=.03 [.00, .05], SRMR=.04, the transactional path from T1 irritability 🡪 T2 psychological control 🡪 T3 irritability only achieved marginal significance (Estimate=.16, SE=.09, *p*=.07 and Estimate=.13, SE=.07, *p*=.06, respectively). The cross-lagged paths from T1 irritability to T2 anxiety (Estimate=.29, SE=.08, *p*<.001), and from T2 irritability to T3 anxiety (Estimate=.23, SE=.11, *p*=.047) remained significant (Figure S2B).

**References**

Achenbach, T. M. (1991). *Manual for the Child Behavior Checklist/4-18 and 1991 profile.*University of Vermont, Department of Psychiatry.

Beck, A. T., Steer, R. A., & Carbin, M. G. (1988). Psychometric properties of the Beck Depression Inventory: Twenty-five years of evaluation. *Clinical Psychology Review*, *8*(1), 77–100. https://doi.org/10.1016/0272-7358(88)90050-5

Curran, P. J., & Bauer, D. J. (2011). The disaggregation of within-person and between-person effects in longitudinal models of change. *Annual Review of Psychology*, *62*, 583-619. <https://doi.org/10.1146/annurev.psych.093008.100356>

Enders, C. K. (2013). Dealing with missing data in developmental research. *Child Development Perspectives*, *7*(1), 27-31. <https://psycnet.apa.org/doi/10.1111/cdep.12008>

Gau, S. S., Shang, C. Y., Liu, S. K., Swanson, J. M., & Tu, C. L. (2008). Psychometric properties of the Chinese version of the Swanson, Nolan, and Pelham, version IV scale - parent form. *International journal of methods in psychiatric research 17,* 35-44.

van Ginkel, J. R., Linting, M., Rippe, R. C., & van der Voort, A. (2020). Rebutting existing misconceptions about multiple imputation as a method for handling missing data. *Journal of Personality Assessment*, *102*(3), 297-308. <https://doi.org/10.1080/00223891.2018.1530680>

Hamaker, E. L., Kuiper, R. M., & Grasman, R. P. (2015). A critique of the cross-lagged panel model. *Psychological Methods*, *20*(1), 102-116. <https://psycnet.apa.org/doi/10.1037/a0038889>

Hong, S., & Lynn, H. S. (2020). Accuracy of random-forest-based imputation of missing data in the presence of non-normality, non-linearity, and interaction. *BMC Medical Research Methodology, 20*(1), 1-12. <https://doi.org/10.1186/s12874-020-01080-1>

Jakobsen, J. C., Gluud, C., Wetterslev, J., & Winkel, P. (2017). When and how should multiple imputation be used for handling missing data in randomised clinical trials–a practical guide with flowcharts. *BMC Medical Research Methodology*, *17*(1), 162. <https://doi.org/10.1186/s12874-017-0442-1>

Mulder, J. D., & Hamaker, E. L. (2021). Three extensions of the random intercept cross-lagged panel model. *Structural Equation Modeling: A Multidisciplinary Journal*, *28*(4), 638–648. https://doi.org/10.1080/10705511.2020.1784738

Shah, A. D., Bartlett, J. W., Carpenter, J., Nicholas, O., & Hemingway, H. (2014). Comparison of random forest and parametric imputation models for imputing missing data using MICE: a CALIBER study. *American Journal of Epidemiology, 179*(6), 764-774. <https://doi.org/10.1093/aje/kwt312>

Stekhoven, D. J. (2013). missForest: Nonparametric missing value imputation using Random Forest. R package version 1.4.

Swanson, J. M. (1992). *School-based assessments and interventions for ADD students.* Irvine, CA: K.C. Press.

Waljee, A. K., Mukherjee, A., Singal, A. G., Zhang, Y., Warren, J., Balis, U., ... & Higgins, P. D. (2013). Comparison of imputation methods for missing laboratory data in medicine. *BMJ Open*, *3*(8), e002847. <http://dx.doi.org/10.1136/bmjopen-2013-002847>

| **Table S1** |  |  |  |  |  |  |  |  |  |  |  |  |
| --- | --- | --- | --- | --- | --- | --- | --- | --- | --- | --- | --- | --- |
| *Comparisons of Sample Characteristics and Study Variables between the Analytic Sample and the Excluded Sample* | | | | | | | | | | | | |
|  | **Analytic sample (*n* = 285)** | | | |  | **Excluded sample (*n* = 454)** | | | |  | **Comparison** | |
| *Sample characteristics* | Mean | *SD* | Min | Max |  | Mean | *SD* | Min | Max |  | *t*/χ^2^ | *p* |
| Child’s age (years; continuous) | 9.9 | 0.6 | 8.8 | 11.4 |  | 10.2 | 0.5 | 8.8 | 11.4 |  | 7.3 | <0.001 |
| Child’s age (grade), *n* (%) |  |  |  |  |  |  |  |  |  |  | 60.96 | <0.001 |
| 4^th^ grade | 141 | 49.5 |  |  |  | 98 | 21.6 |  |  |  |  |  |
| 5^th^ grade | 144 | 50.5 |  |  |  | 356 | 78.4 |  |  |  |  |  |
| Mother’s age (years; continuous) | 40.1 | 4.7 | 29 | 53 |  | 40 | 5 | 23 | 54 |  | -0.1 | 0.92 |
| Child’s sex (female), *n* (%) | 145 | 50.9 |  |  |  | 241 | 53.1 |  |  |  | 0.26 | 0.61 |
| Living arrangement, *n* (%) |  |  |  |  |  |  |  |  |  |  | 1.98 | 0.16 |
| Living with both parents | 252 | 88.4 |  |  |  | 282 | 84.2 |  |  |  |  |  |
| Single-parent family and others | 33 | 11.6 |  |  |  | 53 | 15.8 |  |  |  |  |  |
| Mother’s education level, *n* (%) |  |  |  |  |  |  |  |  |  |  | 1.84 | 0.40 |
| Junior high or below | 41 | 14.6 |  |  |  | 51 | 15.5 |  |  |  |  |  |
| High school | 110 | 39.1 |  |  |  | 143 | 43.6 |  |  |  |  |  |
| College or above | 130 | 46.3 |  |  |  | 134 | 40.9 |  |  |  |  |  |
| Monthly family income, *n* (%) |  |  |  |  |  |  |  |  |  |  | 1.71 | 0.43 |
| Low income (below 1,524 USD) | 98 | 35.8 |  |  |  | 130 | 41.0 |  |  |  |  |  |
| Mid income (1,524 to 3,049 USD) | 128 | 46.7 |  |  |  | 136 | 42.9 |  |  |  |  |  |
| High income (above 3,049 USD) | 48 | 17.5 |  |  |  | 51 | 16.1 |  |  |  |  |  |
| Informant, *n* (%) |  |  |  |  |  |  |  |  |  |  | 0.54 | 0.76 |
| Mothers | 201 | 70.5 |  |  |  | 234 | 70.1 |  |  |  |  |  |
| Fathers | 66 | 23.2 |  |  |  | 74 | 22.2 |  |  |  |  |  |
| Other primary caretakers | 18 | 6.3 |  |  |  | 26 | 7.8 |  |  |  |  |  |
| *Study variables* | Mean | *SD* | Min | Max |  | Mean | *SD* | Min | Max |  | *t*/χ2 | *p* |
| IRR1 | 0.27 | 0.40 | 0 | 2 |  | 0.29 | 0.47 | 0 | 2 |  | 0.51 | 0.61 |
| IRR2 | 0.25 | 0.39 | 0 | 1.67 |  | 0.24 | 0.40 | 0 | 2 |  | -0.31 | 0.76 |
| IRR3 | 0.26 | 0.41 | 0 | 2 |  | 0.30 | 0.47 | 0 | 2 |  | 0.78 | 0.43 |
| ANX1 | 0.24 | 0.29 | 0 | 1.38 |  | 0.24 | 0.28 | 0 | 1.5 |  | -0.11 | 0.91 |
| ANX2 | 0.21 | 0.27 | 0 | 1 |  | 0.22 | 0.26 | 0 | 1.13 |  | 0.39 | 0.69 |
| ANX3 | 0.21 | 0.29 | 0 | 1.75 |  | 0.23 | 0.29 | 0 | 1.5 |  | 0.52 | 0.61 |
| PPC1 | 1.83 | 0.52 | 1 | 3.4 |  | 1.93 | 0.56 | 1 | 5 |  | 1.98 | 0.05 |
| PPC2 | 1.91 | 0.66 | 1 | 5 |  | 1.83 | 0.49 | 1.1 | 2.6 |  | -0.65 | 0.52 |
| PPC3 | 1.94 | 0.80 | 1 | 4.8 |  | 1.85 | 0.47 | 1 | 3.1 |  | -0.97 | 0.33 |
| CPC1 | 2.43 | 0.81 | 1 | 5 |  | 2.54 | 0.94 | 1 | 5 |  | 1.52 | 0.13 |
| CPC2 | 2.41 | 0.88 | 1 | 5 |  | 2.51 | 0.93 | 1 | 5 |  | 1.05 | 0.29 |
| CPC3 | 2.46 | 0.90 | 1 | 5 |  | 2.40 | 0.86 | 1 | 5 |  | -0.69 | 0.49 |
| *Note.* Data prior to imputation are shown here. IRR = child irritability, ANX = child anxiety, PPC = parent-rated parent psychological control, CPC = child-rated parent psychological control. | | | | | | | | | | | | |

**Figure S1**

*Density Plots of Key Study Variables Before and After Imputation*

**
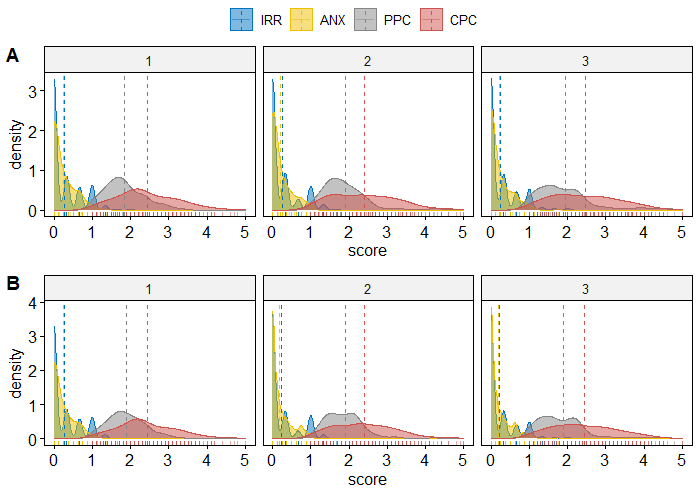
**

*Note.* (A) Before imputation. (B) After imputation using the missForest package in R. Dotted lines denote the means of study variables at each time point. IRR = child irritability, ANX = child anxiety, PPC = parent-rated parent psychological control, CPC = child-rated parent psychological control.

**Figure S2**

*Transactional Associations between Child Irritability, Child Anxiety, and Child-Rated Parent Psychological Control with further adjustments for parent sad mood and child depressive and ADHD symptoms.*


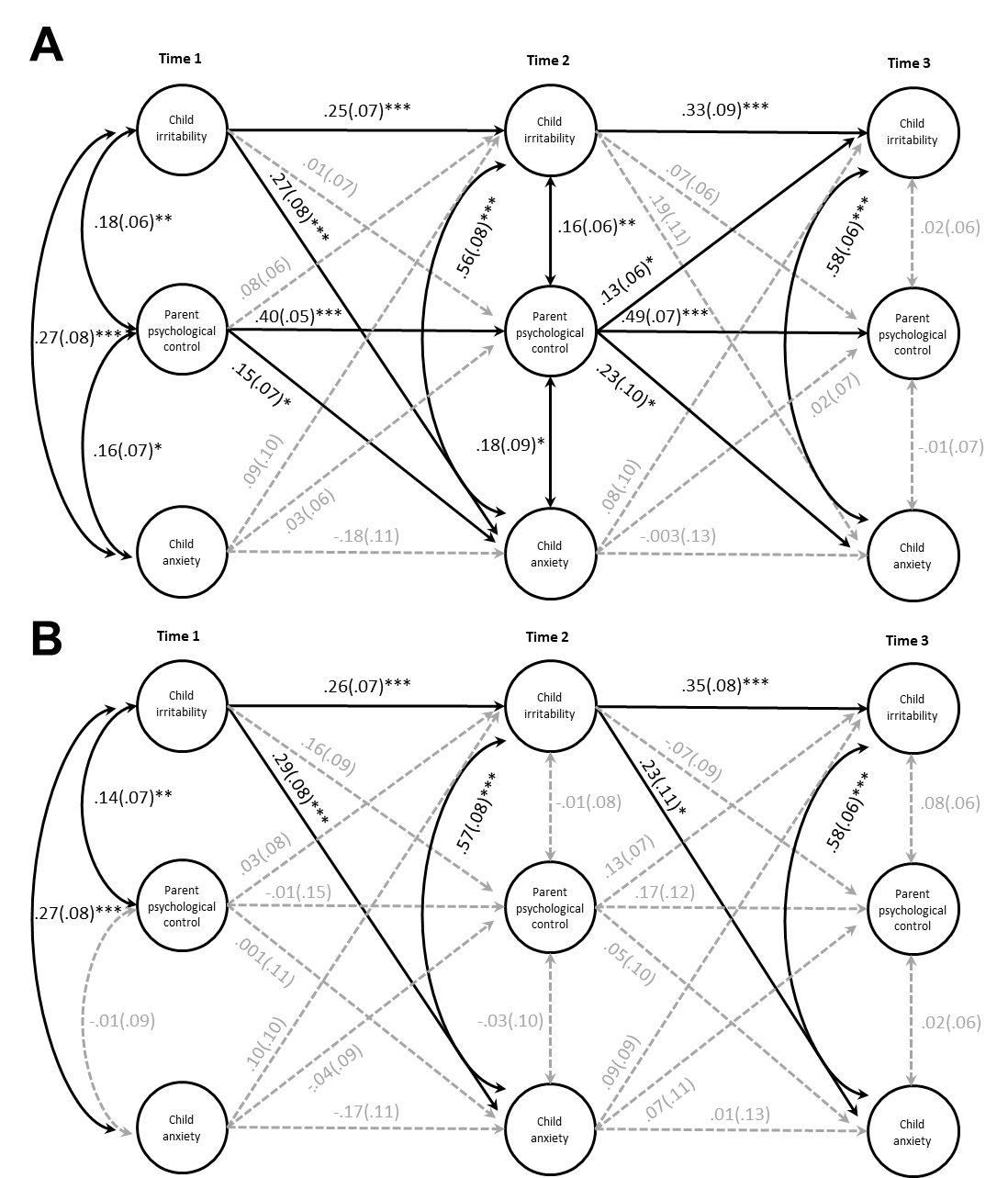


*Note.* (A) Parenting data from parents. The variances of the random intercepts for child irritability and parent-rated parent psychological control were constrained to zero. (B) Parenting data from children. The variances of the random intercept for child irritability were constrained to zero. Significant paths are denoted by solid black lines; non-significant paths are denoted by dashed grey lines. To avoid clutter, the paths estimating the between-person random intercepts were estimated but are not visualised here. Standardized estimates (standard errors) are shown.

**p*≤.05, ***p*≤.01, ****p*≤.001.
